# Factors Affecting the Survival of Metastatic Breast Cancer Patients Treated with CDK 4/6 Inhibitors

**DOI:** 10.3390/medicina61071279

**Published:** 2025-07-16

**Authors:** Zehra Sucuoğlu İşleyen, Harun Muğlu, Zeynep Alaca Topçu, Mehmet Beşiroğlu, Ayşe İrem Yasin, Atakan Topçu, Melih Şimşek, Mesut Şeker, Hacı Mehmet Türk

**Affiliations:** 1 Department of Medical Oncology, Bezmialem Vakif University Hospital, 34093 Istanbul, Turkey; ayseiremyasin@gmail.com (A.İ.Y.); atakantopcu@hotmail.com (A.T.); mdmelih@gmail.com (M.Ş.); drmesutseker@gmail.com (M.Ş.); hmehmetturk@gmail.com (H.M.T.); 2 Department of Medical Oncology, Medipol University Hospital, 34214 Istanbul, Turkey; hm1635@hotmail.com; 3 Department of Medical Oncology, Istanbul Medeniyet University Hospital, 34722 Istanbul, Turkey; drzeynepalaca@gmail.com (Z.A.T.); mbesiroglu@yahoo.com.tr (M.B.)

**Keywords:** metastatic breast cancer, CDK 4/6 inhibitors, ribociclib, palbociclib

## Abstract

*Background and Objective:* We aim to determine the efficacy and the factors associated with the effectiveness of first-line CDK4/6i (ribociclib or palbociclib) treatment in HR-positive, HER2-negative MBC patients. *Materials and Methods:* This is a retrospective, cross-sectional, and descriptive study. Ninety patients with metastatic breast cancer receiving CDK 4/6i treatment from three different oncology clinics were included in this study. *Results:* Of the patients, 56 (62.2%) received ribociclib, and 34 (37.8%) were administered palbociclib. There was no significant difference between the groups regarding age, gender, comorbidities, ECOG performance status, or menopausal status (*p* > 0.05). The cut-off values for ER, PR, and Ki-67 levels were determined via ROC curve analysis. These values were found to be 80% for ER levels, 50% for PR levels, and 30% for Ki-67 levels. PFS was significantly longer for patients with ER levels greater than 80% and Ki-67 expression levels less than 30% according to multivariate analysis. Among the patients included in our study, the median PFS was 22.41 months for the patients with Ki-67 levels of 30% and above, while the median PFS was 17.24 months for the patients with ER levels of 80% and below. Among the patients with a combined ER of 80% or less and a Ki-67 of 30% or more, the median PFS was 12.42 months (*p* < 0.001). *Conclusions:* This study demonstrates that CDK4/6i therapies led to longer PFS among patients with ER levels greater than 80% and Ki-67 expression levels less than 30%. It is essential to determine which patient group benefits more from first-line CDK4/6is therapy.

## 1. Introduction

Breast cancer is the most common malignancy diagnosed in women worldwide and the leading cause of mortality [1]. Approximately 70% of breast cancer is hormone receptor (HR)-positive and human epidermal growth factor receptor 2 (HER2)-negative. HR-positive, HER2-negative breast cancers have better prognoses than other types [2]. Although significant improvements in survival have been seen with current systemic therapies, metastatic breast cancer (MBC) remains incurable [3]. The cornerstone of initial treatment for metastatic HR-positive, HER2-negative breast cancer is endocrine therapy [4].

Cyclin-dependent kinases (CDKs) play a critical role in regulating the cell cycle. CDK4 and CDK6 are the key regulators of the G1-S phase transition, driving cell proliferation. In HR-positive breast cancer, dysregulation of these CDKs contributes to uncontrolled tumor growth. CDK 4/6 inhibitors (CDK4/6i) function by binding to the ATP-binding pocket of CDK4 and CDK6, thereby preventing their interaction with cyclin D and subsequent phosphorylation of the retinoblastoma protein (Rb). This ultimately leads to cell cycle arrest and inhibits tumor growth [5]. Adding CDK4/6i (e.g., ribociclib, palbociclib, or abemaciclib) to endocrine therapy has proven clinically beneficial. Compared to endocrine therapy alone, the combination of CDK4/6i and endocrine therapy led to a significant improvement in progression-free survival (PFS) and overall survival (OS) [6]. In the MONALEESA-2 study, ribociclib demonstrated significant PFS and OS benefits when combined with an aromatase inhibitor (AI) to treat postmenopausal patients with HR-positive, HER2-negative advanced breast cancer [7]. Significant PFS and OS benefits were seen in the MONALEESA-7 study in regard to premenopausal patients and for postmenopausal patients in the MONALEESA-3 study [8,9]. In the PALOMA-2 trial, palbociclib did not show an OS benefit, but there was a significant PFS benefit [10].

Patient characteristics, treatment factors, and tumor biology may play a role in the effectiveness of CDK4/6i. Understanding these factors is crucial to tailor treatment strategies and optimize outcomes for HR-positive, HER2-negative MBC patients [11,12]. Ki-67 is a proliferation marker indicating the percentage of actively dividing cells within a tumor. Higher Ki-67 scores and higher tumor grades generally correlate with aggressive disease, and, in recent studies, PFS was found to be significantly shorter among these patients [11,13]. The impact of estrogen receptor (ER) and progesterone receptor (PR) levels on CDK4/6i efficacy needs to be clarified. While some studies suggest there is no significant association, others indicate potential benefits for patients with higher ER and PR expression [12,13,14].

This study aims to determine the efficacy and the factors associated with the effectiveness of first-line CDK4/6i (ribociclib or palbociclib) in HR-positive, HER2-negative MBC patients.

## 2. Materials and Methods

This is a retrospective, cross-sectional, and descriptive study. The study was performed among patients with MBC who were referred to the medical oncology clinic between December 2019 and January 2024. A total of 90 patients from three different oncology outpatient clinics in Turkey were enrolled.

The inclusion criteria for this research were a confirmed diagnosis of MBC, having HR-positive and HER2-negative primary tumors, and having received first-line CDK4/6i (ribociclib or palbociclib) treatment for metastatic disease. Patients who received chemotherapy or endocrine therapy in an adjuvant or neoadjuvant setting were included in this study. Patients who were under 18 years of age did not undergo treatment response evaluation, or had been undergoing CDK4/6i-based treatment for less than three months, were not included in this study. This study was conducted in accordance with the Declaration of Helsinki and approved by the Bezmialem Vakıf University ethics approval committee (2022/396).

The cut-off values for estrogen receptor (ER), progesterone receptor (PR), and Ki-67 were established using receiver operating characteristic (ROC) curve analysis to maximize the predictive accuracy for clinical outcomes. Consistent with the statistical principles outlined by Frank E. Harrell Jr., we selected cut-off values based on empirical data rather than arbitrary conventions, considering both sensitivity and specificity. The optimal values were determined by identifying the point on the ROC curve with the greatest combined sensitivity and specificity (Youden Index).

The 8th edition TNM staging system was used for tumor staging. Demographic characteristics, clinical and pathological features, treatment responses, the number of medications, and comorbid diseases of the patients were collected. Functional status was measured using the Eastern Cooperative Oncology Group (ECOG) scale.

PFS was defined as the time from initiation of CDK4/6i therapy to the progression of the disease or the last day of follow-up. OS was defined as the time from initiation of CDK4/6i therapy to death or the last day of follow-up.

### Statistical Analysis

Statistical analysis was performed using Statistical Package for Social Sciences version 25 (SPSS, v25). Qualitative variables were described by frequencies and percentages, continuous variables by mean and standard deviation, or median and range. The normal distribution range was determined by the Kolmogorov–Smirnov test. A *T*-test and a Chi-square test were used to describe patient characteristics and to compare subgroups of patients. The factors associated with PFS were assessed using univariate and multivariate logistic regression analysis. Multivariable analysis was performed on data that was statistically significant in univariable analysis. Hazard ratios (HR) were calculated to evaluate the magnitude of the impact of the significant variables with a 95% confidence interval (CI). Survival analysis was performed using the Kaplan–Meier method and log-rank test. The cut-off value for ER, PR, and Ki-67 levels was determined by ROC curve analysis. The statistical significance level was accepted as a *p*-value < 0.05.

## 3. Results

The study included 90 patients with MBC who received first-line CDK4/6i. Of the patients, 56 (62.2%) had received ribociclib, and 34 (37.8%) palbociclib. There was no significant difference between the groups regarding age, gender, comorbidities, ECOG performance status, and menopausal status (*p* > 0.05) (Table 1).

In the present study, the patients using fulvestrant (*n* = 14, 15.6%) with CDK4/6i were those who developed recurrence under adjuvant AI therapy. Among the patients in the study, 39 had non-visceral metastases, and 51 had visceral metastases. PFS was significantly shorter in patients with liver metastases compared with those without. However, no significant relationship was found between other metastasis sites and PFS. Forty-six patients (51.1%) had metastatic disease after undergoing curative treatment for primary breast cancer, while 44 patients were de novo metastatic; there were no significant differences in PFS among these patients (*p*: 0.130) (Table 2).

**Figure 1:**
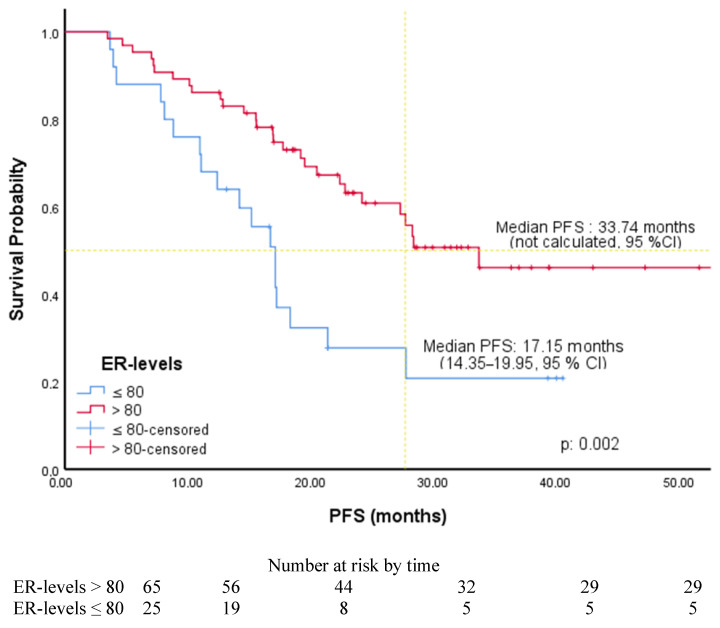
PFS of patients according to ER level.

**Figure 2:**
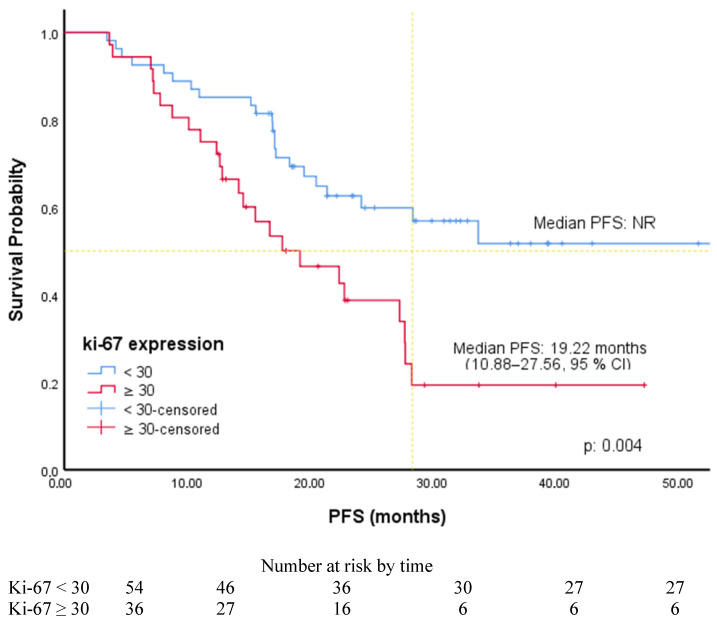
PFS of patients according to ki-67 expression.

**Figure 3:**
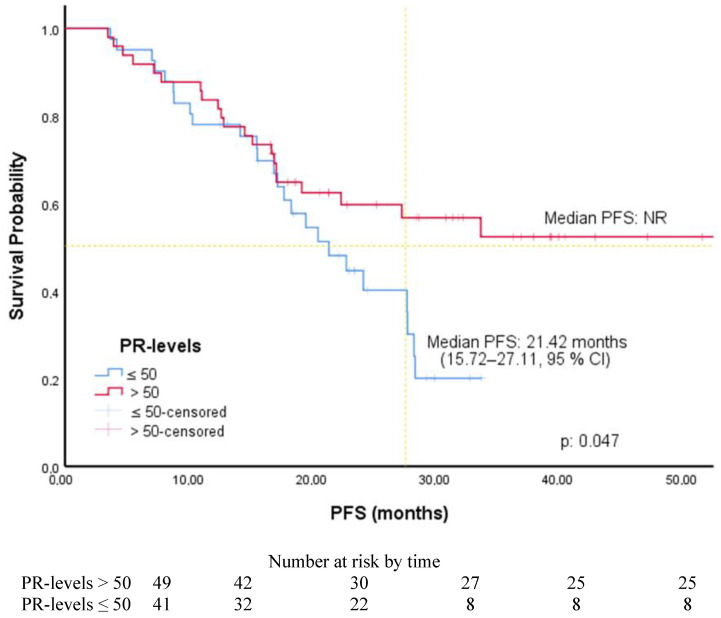
PFS of patients according to PR level.

**Figure 4:**
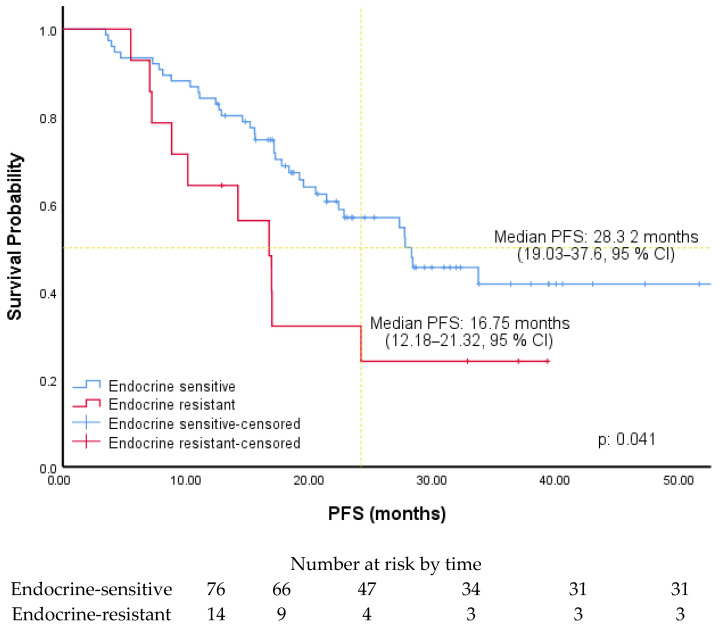
PFS of patients according to ER resistance.

**Figure 5:**
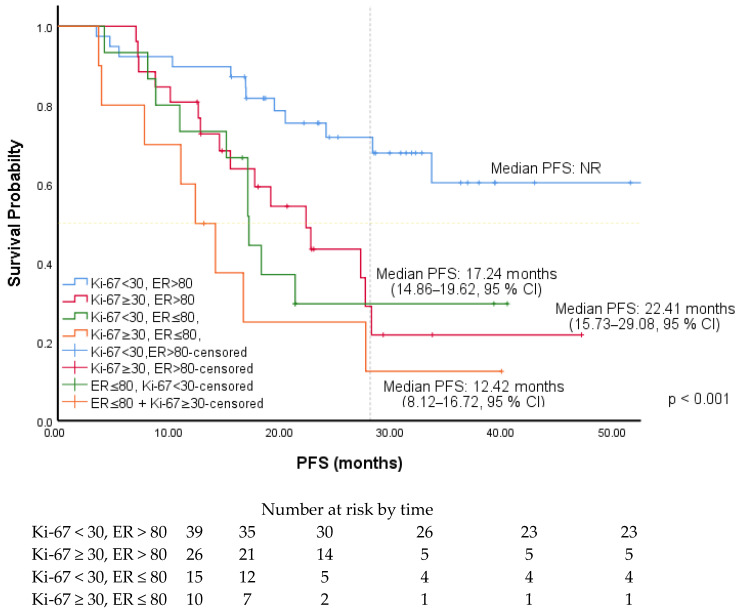
PFS of patients based on both ER level and Ki-67 expression.

The cut-off values for ER, PR, and Ki-67 levels were determined by ROC curve analysis. It was found to be 80% for ER level [AUC: 0.650 (0.537–0.763), *p*: 0.014], 50% for PR level [AUC: 0.624 (0.506–0.741), *p*: 0.044], and 30% for Ki-67 level [AUC: 0.644 (0.529–0.758), *p*: 0.019]. Associations between PFS and patients’ and tumors’ characteristics were investigated using Cox regression analysis. In univariate analysis, PFS was significantly longer in patients with luminal A subtype (HR: 2.28, 95% CI, 1.24–4.21), without liver metastases (HR: 2.23, 95% CI, 1.17–4.24), receiving AI (2.05, 95% CI, 1.01–4.15), with an ER level greater than 80%, a PR level greater than 50% (1.80, 95% CI, 1.0–3.25), and a Ki-67 expression less than 30%. In multivariate analysis, PFS was significantly longer in patients with an ER level greater than 80% (HR: 2.50, 95% CI, 1.36–4.61) and Ki-67 expression was less than 30% (HR: 2.22, 95% CI, 1.22–4.05) (Table 2). The PFS of the patients according to the ER level and ki-67 expression is shown in Figure 1 and Figure 2. Among the patients included in our study, the median PFS was 22.41 months (15.73–29.08, 95% CI) in the patients with only a Ki-67 level of 30% and above, while the median PFS was 17.24 months (14.86–19.62, 95% CI) in the patients with only an ER level of 80% and below. The PFS of the patients according to PR level and endocrine resistance is shown in Figure 3 and Figure 4. In the patients with a combined ER level of 80% or less and a Ki-67 level of 30% or more, the median PFS was 12.42 months (8.12–16.72, 95% CI) (Figure 5).

**Figure 6:**
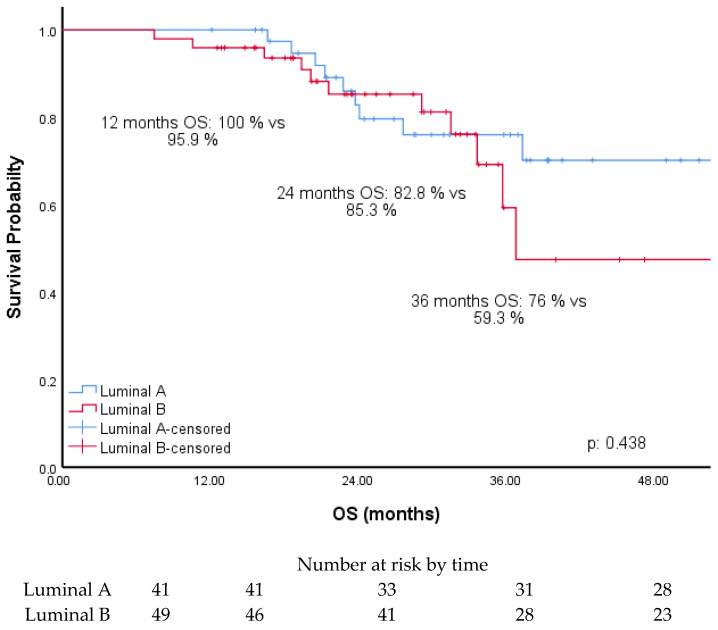
OS of patients according to luminal types.

The median follow-up time was 30 months. The median PFS (mPFS) of all patients was 27.3 months (95% CI, 20.9–33.7). mPFS in patients with luminal-A type was not reached, and mPFS with luminal-B type was 19.2 months (95% CI, 13.1–25.3) (*p*: 0.018). The median OS (mOS) of all patients was not reached. In addition, mOS in patients with luminal A type was not reached, and mOS in patients with luminal B type was 36.8 months (*p*: 0.438). The survival rates of the patients with luminal A and luminal B types were 100% and 95.9% at 12 months, 82.8% and 85.3% at 24 months, and 76% and 59.3% at 36 months, respectively (Figure 6). The PFS of the patients with luminal A and luminal B types were 86.4% and 76.1% at 12 months, 63.8% and 43.1% at 24 months, and 51.8% and 27.4% at 36 months, respectively.

## 4. Discussion

This study evaluated the predictive factors of pathological and clinical characteristics for the prognosis of MBC patients treated with CDK4/6is. Results showed that CDK4/6i therapies led to longer PFS in patients with ER levels over 80% and Ki-67 expression under 30% (*p* < 0.05). Univariate analysis indicated PFS was significantly longer in the luminal A subtype and PR levels greater than 50%. However, multivariate analysis showed that PR levels and luminal subtypes were not associated with prolonged PFS. Similarly, liver metastases were prognostic in univariate analysis but not in multivariate analysis. No significant OS difference was observed between luminal A and B subtypes (*p* = 0.438).

CDK4/6is are the standard first-line treatment for HR-positive and HER2-negative MBC. However, no strong predictors exist for tumor response to CDK4/6i. It is known that the luminal subtype alone is considered insufficient for guiding treatment decisions [15]. ER and PR positivity and HER2 status are the key prognostic factors. Additionally, higher levels of ER and PR are associated with longer PFS and OS in MBC [16]. However, it is not clear to what extent ER and PR levels predict the response to CDK4/6i. ER positivity is the best predictor of CDK4/6i and hormone therapy in a large pooled US Food and Drug Administration (FDA) analysis. However, the relationship between ER levels and response rate has not been investigated, and PR status was not considered as a predictive marker in this study [6]. In a study conducted by Ge et al. [17], the median ER level of patients using CDK 4/6 was 95%, the PR level was 62.5% and the ki-67 level was 30%. In addition, a significant association was found between increased levels of the receptor and PFS [18]. In our study, patients with ER levels > 80% had significantly longer PFS than those with ER levels < 80% (33.7 vs. 17.1 months, *p* = 0.002). The SONIA trial compared two treatment strategies: first-line CDK4/6 inhibitor plus nonsteroidal aromatase inhibitor (NSAI) followed by second-line fulvestrant versus first-line NSAI followed by CDK4/6 inhibitor plus fulvestrant upon progression. No significant differences were observed in second progression-free survival (PFS2) or OS between the two arms, raising questions about the optimal timing of CDK4/6 inhibitor use in terms of cost-effectiveness [19].

In an analysis of the MONARCH-2 and MONARCH-3 studies, shorter PFS was observed in patients receiving AI monotherapy with either PR-negative or high-grade tumors. However, there was no significant association between PFS and these factors in those receiving abemaciclib with endocrine therapy [15]. Cristofanilli et al. investigated the factors that predicted the long-term benefit of palbociclib and fulvestrant in their analysis of the PALOMA-3 study [16]. They showed that a high level of PR expression was associated with prolonged clinical benefit [16]. In a study conducted by Jia et al., patients with PR below 10% were considered to be low, and a significant relationship was found with PFS [18]. In our study, the number of patients with PR below 10% was small. In our study, ROC curve analysis identified a PR expression threshold of 50% as the optimal cut-off. Although PR level and luminal subtype were significant in univariate analysis, they lost significance in the multivariate model. Collinearity diagnostics showed that all VIF values were below 2.5, suggesting that multicollinearity was not a major issue. This loss of significance may be due to overlapping biological roles of the ER, PR, and luminal subtypes, where the ER’s dominant predictive effect may mask the contribution of PR [16]. The limited sample size may also have reduced the power to detect independent associations. Overall, the predictive value of PR expression in response to CDK4/6 inhibitors remains uncertain. Notably, one study reported no association between PR levels and disease recurrence [20].

The choice of therapy in MBC is based on prognostic factors such as histological tumor type, tumor size, nodal status, grade, age, HR status, and ki-67 expression. Ki-67 is a nuclear protein identified in 1983 and expressed during all cell cycle phases except G0. The prognostic value of ki-67 in breast cancer has been widely investigated [21]. Clinical and pathological responses to neoadjuvant chemotherapy in breast cancer patients were better in those with higher Ki-67 levels [22]. The MonarchE study showed that the patients with high ki-67 expression achieved a statistically significant improvement in invasive disease-free survival (iDFS) with abemaciclib in combination with adjuvant endocrine therapy [23]. Although studies have shown that high Ki-67 levels are associated with better chemotherapy response in MBC, it is known that Ki-67 is a poor prognostic marker [24]. A meta-analysis by Azambuja et al. confirmed that high Ki-67 levels confer a worse DFS and OS in breast cancer [25]. Wang et al. identified Ki-67 as a significant predictor of PFS in patients with MBC receiving palbociclib, which is consistent with the findings of Lee et al. [26,27]. Our study showed that PFS was significantly shorter in patients with Ki-67 expression ≥ 30%. mPFS was not reached in the patients with Ki-67 expression < 30%, while mPFS was 19.2 months in the patients with Ki-67 expression ≥ 30% (*p*: 0.004). Several studies, consistent with our findings, have reported that high Ki-67 expression adversely impacts PFS, while no significant association with PR levels was observed [12,20]. Although ER, PR and Ki-67 are standard biomarkers, their role as predictive tools for CDK4/6i response is still being investigated. Our findings suggest that quantifying these markers using ROC-based thresholds could provide additional value in the clinical decision-making process.

The impact of HER2 status on treatment outcomes in HR-positive HER2-negative breast cancer patients is a new topic of discussion. Although HER2-low (IHC 1+ or 2+/ISH–) and HER2-zero (IHC 0) tumors are both classified as HER2-negative, accumulating evidence suggests that they may differ biologically and clinically. In our study, HER2 status did not show a significant association with PFS. However, our study was not specifically powered to detect subtle differences between HER2-low and HER2-zero subgroups. Studies investigating the effect of HER2 status on treatment have conflicting results. In a study investigating the effect of HER2 status on treatment response in patients using CDK 4/6i, no significant effect of HER2 status on PFS was shown [28]. A recent study by Sharaf et al. (2023) reported that HER2-low status was independently associated with worse progression-free survival compared to HER2-zero disease in patients receiving ribociclib (mPFS 17.3 vs. 22.2 months, *p* = 0.0039) [29]. This emerging distinction between HER2-low and HER2-zero disease represents a promising area for future research and therapeutic stratification.

The overall mPFS in our study was 27.3 months and 19.2 months in the patients with the luminal-B subtype. In the MONALEESA-7 study, mPFS reached 23.8 months in premenopausal patients with ribociclib, while in the MONALEESA-3 study, mPFS in postmenopausal patients was 20.5 months. In the MONALEESA-7 study, some of the patients had received chemotherapy before receiving CDK4/6i in the advanced stage, while in the MONALEESA-3 study, they had received hormone therapy. The median progression-free survival (mPFS) observed in our study is consistent with previously published data on CDK4/6 inhibitor efficacy in the first-line treatment of hormone receptor–positive metastatic breast cancer. In a recent meta-analysis by Gao et al., which pooled data from phase III clinical trials evaluating CDK4/6 inhibitors in this setting, the reported mPFS was 28 months, closely aligning with our findings [6]. Similarly, randomized phase III trials such as PALOMA-2 and MONALEESA-2 demonstrated mPFS durations of 27.6 and 25.3 months, respectively, among patients who were naïve to systemic therapy in the metastatic setting [30,31]. Our data showed that PFS was shorter in patients with liver metastasis in univariate analysis. However, this was not statistically significant on multivariate analysis, possibly because of the small number of patients with liver metastasis.

In our study, PFS was significantly shorter among patients receiving fulvestrant with CDK4/6i. These patients had previously progressed while receiving adjuvant aromatase inhibitor (AI) therapy and were therefore classified as endocrine resistant. Given the small sample size in the fulvestrant subgroup (*n* = 14), the results should be considered exploratory and interpreted with caution. As shown in clinical studies, the contribution of CDK4/6i to PFS is less in endocrine-resistant patients than in endocrine-sensitive patients [26].

In the PALOMA-2 and MONALEESA-3 studies, dose reductions were required in 36% and 37.9% of the patients, respectively, while this rate was 50% in our study [30,32]. Similarly to the PALOMA-2 study, no significant difference in PFS was observed in patients with dose reduction in CDK4/6i treatment. The rate of all-grade adverse events in the PALOMA-2 study was 98.9%, whereas 99% in the MONALEESA-3 study. In our study, any grade adverse events occurred in 93.3% of the patients [30,32].

This study has some limitations. Our research is a retrospective study with a limited sample size. Additionally, pathological assessments were conducted across three different pathology centers. Furthermore, the study population included endocrine-resistant patients. Although the sample size was limited, our results are consistent with recent trials and provide data from a real-world setting. Nonetheless, caution should be exercised in interpreting these associations due to limited statistical power. However, the present study has multiple strengths. The patients who were admitted to three different oncology outpatient clinics were included in the study, and our findings reflect real-world patient populations. Moreover, none of the patients received chemotherapy or endocrine therapy in the metastatic setting prior to CDK4/6 inhibitor initiation.

## 5. Conclusions

CDK4/6i have broken new ground in the treatment of HR-positive, HER2-negative MBC by providing significant contributions to PFS and OS. However, these agents are also associated with both pharmacologic and financial toxicities. Therefore, it is essential to determine which patient subgroups derive the greatest benefit from first-line CDK4/6is. Our results showed that CDK4/6i therapies prolonged PFS in patients with ER levels greater than 80% and Ki-67 expression less than 30%. Additionally, luminal type, liver metastasis, endocrine resistance and PR level also provided a statistically non-significant association with PFS. Comprehensive studies will ensure which cost-effective treatments may lead to maximum benefit for the patients with minimum toxicity.

## Figures and Tables

**Table 1:** Baseline characteristics of the patients.

Characteristics	All Patients, *n* (%)	Luminal A, *n* (%)	Luminal B, *n* (%)	*p*-Value
Age				
<65 years	63 (70%)	31 (70.5%)	32 (69.6%)	
≥65 years	27 (30%)	13 (29.5%)	14 (30.4%)	0.927
Sex				
Women	87 (96.7%)	43 (97.7%)	44 (95.7%)	
Men	3 (3.3%)	1 (2.3%)	2 (4.3%)	0.584
Smoking				
Yes	15 (16.7%)	8 (18.2%)	7 (15.2%)	
No	75 (83.3%)	36 (81.8%)	39 (84.8%)	0.706
Comorbidities				
DM	19 (21.1%)	10 (22.7%)	9 (19.6%)	0.713
HT	29 (32.2%)	12 (27.3%)	17 (37%)	0.326
CAD	9 (10%)	4 (9.1%)	5 (10.9%)	0.779
ECOG-PS				
0–1	79 (87.8%)	39 (88.6%)	40 (87%)	
2	11 (12.2%)	5 (11.4%)	6 (13%)	0.808
Polypharmacy				
Yes	19 (21.1%)	11 (25%)	8 (17.4%)	
No	71 (78.9%)	33 (75%)	38 (82.6%)	0.337
Histology				
Ductal carcinoma	74 (82.2%)	35 (79.5%)	39 (84.8%)	
Lobular carcinoma	12 (13.3%)	7 (15.9%)	5 (10.9%)	
Others	4 (4.4%)	2 (4.5%)	2 (4.3%)	0.777
Grade				
1–2	63 (70%)	41 (93.2%)	22 (47.8%)	
3	27 (30%)	3 (6.8%)	24 (52.2%)	<0.001
Menopausal Status				
Premenopause	41 (45.6%)	22 (50%)	19 (41.3%)	
Postmenopause	49 (54.4%)	22 (50%)	27 (58.7%)	0.408
Breast cancer				
De novo	44 (48.9%)	24 (54.5%)	20 (43.5%)	
Recurrent	46 (51.1%)	20 (45.5%)	26 (56.5%)	0.294
Metastatic sites				
Visceral	51 (56.7%)	22 (50%)	29 (63%)	
Non-visceral	39 (43.3%)	22 (50%)	17 (37%)	0.212
Metastatic sites				
Bone	79 (87.8%)	41 (93.2%)	38 (82.6%)	0.126
Liver	16 (17.8%)	7 (15.9%)	9 (19.6%)	0.650
Lung	29 (32.2%)	14 (31.8%)	15 (32.6%)	0.936
Cranial	3 (3.3%)	0	3 (6.5%)	0.085
Lymph nodes, distant	23 (25.6%)	9 (20.5%)	14 (30.4%)	0.278
Number of metastatic sites				
1	47 (52.2%)	24 (54.5%)	23 (50%)	
2	22 (24.4%)	10 (22.7%)	12 (26.1%)	
≥3	21 (23.3%)	10 (22.7%)	11 (23.9%)	0.902
Prior therapy for EBC				
Surgery	54 (60%)	24 (54.5%)	30 (65.2%)	0.302
Chemotherapy	46 (51.1%)	20 (45.5%)	26 (56.5%)	0.294
Endocrine therapy	46 (51.1%)	20 (45.5%)	26 (56.5%)	0.294
Endocrine therapy during CDK4/6i				
Aromatase inhibitors	76 (84.4%)	38 (86.4%)	38 (82.6%)	
Fulvestrant	14 (15.6%)	6 (13.6%)	8 (17.4%)	0.623
Dose Reduction				
Yes	45 (50%)	21 (47.7%)	24 (52.2%)	
No	45 (50%)	23 (52.3%)	22 (47.8%)	0.673

**Table 2:** Univariate and multivariate analyses of PFS.

	Univariate Analysis			Multivariate Analysis		
Variables	HR	95% CI	*p*-Value	HR	95% CI	*p*-Value
Age						
<65 years (R)	0.60	0.29–1.25	0.174			
≥65 years						
Menopausal Status						
Premenopause	1.08	0.60–1.94	0.787			
Postmenopause						
Smoking						
Yes	0.65	0.28–1.56	0.342			
No (R)						
Polypharmacy						
Yes	1.14	0.56–2.30	0.714			
No (R)						
Grade						
1–2	1.75	0.96–3.19	0.069			
3						
Luminal type						
Luminal A	2.28	1.24–4.21	0.008	1.39	0.52–3.67	0.511
Luminal B						
Breast cancer						
De novo	1.57	0.87–2.83	0.130			
Recurrent						
Metastatic sites						
Non-visceral (R)	0.93	0.52–1.67	0.820			
Visceral						
Bone metastasis	1.50					
Yes		0.59–3.80	0.392			
No (R)						
Liver metastasis						
Yes	2.23	1.17–4.24	0.014	1.55	0.76–3.16	0.227
No (R)						
Lung metastasis						
Yes	0.62	0.31–1.22	0.163			
No (R)						
Cranial metastasis						
Yes	1.87	0.45–7.75	0.387			
No (R)						
Lymph nodes, distant						
Yes	0.95	0.48–1.87	0.878			
No (R)						
Endocrine therapy during CDK 4/6i						
Aromatase inhibitors (R)	2.05	1.01–4.15	0.045	1.98	0.96–4.08	0.064
Fulvestrant						
CDK 4/6i adverse events						
Yes	1.71	0.41–7.06	0.458			
No (R)						
Dose Reduction						
Yes	0.92	0.51–1.64	0.780			
No (R)						
ER (ROC)						
≤80	2.48	1.36–4.52	0.003	2.50	1.36–4.61	0.003
>80 (R)						
PR (ROC)						
≤50	1.80	1.0–3.25	0.05	1.69	0.92–3.11	0.088
>50 (R)						
Ki-67 (ROC)						
<30 (R)	2.31	1.28–4.14	0.005	2.22	1.22–4.05	0.009
≥30						
Her-2 positivity	1.05	0.69–1.60	0.810			
ECOG						
1–2 (R)	1.20	0.47–3.06	0.696			
3						
Number of metastatic sites						
1 (R)						
2	1.16	0.59–2.43	0.695			
≥3	1.27	0.63–2.53	0.503			

(CDK 4/6i: cyclin-dependent kinase 4/6 inhibitor, ECOG-PS: Eastern Cooperative Oncology Group-Performance Status, ER: estrogen receptor, HR: hazard ratio, PR: progesterone receptor).

## Data Availability

The original contributions presented in this study are included in the article. Further inquiries can be directed to the corresponding author.
